# A High-Resolution Multipath Delay Measurement Method Using KFSC-WRELAX Algorithm

**DOI:** 10.3390/s24154968

**Published:** 2024-07-31

**Authors:** Yu Dong, Zhizhong Zhang

**Affiliations:** School of Electronics & Information Engineering, Nanjing University of Information Science & Technology, Nanjing 210044, China; 202212180045@nuist.edu.cn

**Keywords:** m-sequence, Kalman filtering, sliding correlation, WRELAX algorithm, channel measurement

## Abstract

Given the challenges associated with the low accuracy, complexity of the equipment, and poor interference resistance observed in current wireless multipath channel measurements, this study introduces a novel algorithm called KFSC-WRELAX. This algorithm integrates techniques involving pseudorandom noise (PN) sequences, Kalman filtering (KF), sliding correlation, and weighted Fourier transform combined with the RELAXation (WRELAX) algorithm. An m-sequence is employed as the probing sequence for channel detection. The effectiveness of the KFSC-WRELAX algorithm is demonstrated through both simulation experiments and corridor testing, showing that it can accurately determine the delays in various paths with robust performance at a signal-to-noise ratio (SNR) of −5 dB or higher.

## 1. Introduction

Satellite communication and navigation are technologies that utilize satellites to relay information and provide global positioning and navigation services [1]. Satellite communication enables global telephony, broadcasting, internet access, and military communications, while satellite navigation systems such as GPS and BeiDou offer global positioning and navigation services, which are extensively used in transportation, aviation, military, and resource exploration. Consequently, satellites have become primary targets in electronic warfare on modern battlefields [2]. To maintain communication and navigation capabilities, an auxiliary terminal positioning system is required that can ensure command centers are informed of unit positions even when satellite communication and navigation fail, thereby enhancing strategic deployment capabilities. Thus, it is necessary to select suitable auxiliary positioning technologies based on environmental conditions and precision requirements.

Currently, auxiliary positioning technologies primarily include Wi-Fi positioning, geomagnetic positioning, inertial navigation, and feature information matching [3,4]. Wi-Fi positioning [5], which determines location based on nearby Wi-Fi signal strength, suffers from severe signal interference and network discontinuity: geomagnetic positioning [6,7] uses the Earth’s magnetic field but is susceptible to electromagnetic interference; visual positioning [8] uses cameras and computer vision to determine terminal location through image recognition and matching, but environmental changes significantly affect its accuracy; and inertial navigation systems estimate device movement and directional changes using accelerometers and gyroscopes but typically accumulate drift over time, preventing their standalone use [9]. Compared to these technologies, a more stable feature fingerprint is required for real-time positioning. Channel characteristics, as relatively stable feature information, can serve as fingerprint information for confirming positions. Among various channel characteristics, multipath delay information is more robust than channel gain due to power variations between transmitters and receivers, making it more suitable for fingerprint-based positioning.

Presently, there are various delay estimation algorithms. Reference [10] described a method using the FZC (Frank–Zadoff–Chu) sequence combined with sliding correlation on a software radio platform, which struggled with a low signal-to-noise ratio and simultaneous measurements by multiple devices. The MODE-WRELAX algorithm proposed in Reference [11] did not handle noise effectively, resulting in significant estimation errors at low SNRs. Reference [12] also employed a wavelet thresholding method for denoising received signals and proposed the GSCC-WRELAX method for mountainous environments, which had a low delay resolution and was unsuitable for complex urban settings. Reference [13] used machine learning for channel measurements, which was impractical for portable measurements. This paper addresses these issues by using an m-sequence as the probing sequence, suitable for simultaneous measurements by multiple devices, thus enhancing measurement efficiency; it combines Kalman filtering with sliding correlation to improve SNR, ensuring system accuracy at low SNR, and employs the WRELAX algorithm to extract multipath parameters from the correlated signals. Simulation results demonstrate that the estimated average error can be reduced to below 1 ns at a SNR of −5 dB.

## 2. System Architecture

**Figure 1:**
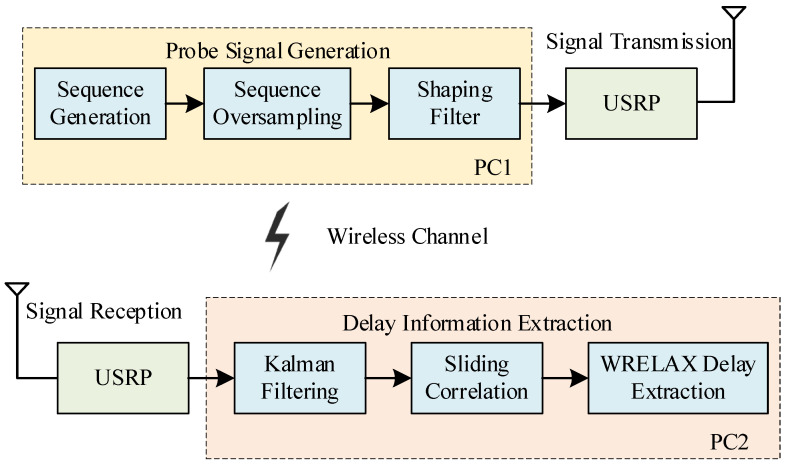
The measurement simulation system structure mainly consists of three parts: signal transmission processing, wireless channel, and signal reception decomposition.

Wireless channel multipath delay measurement primarily aims to determine a channel’s multipath delays. In wireless communications, the signals reaching the receiver are superpositions of signals that have traveled different paths using various time delays. These superposed signals exhibit a time delay spread in the time domain relative to the original signal. Wireless channel multipath delay measurement is intended to measure the delays of the superposed signals along each path, that is, the channel’s delay distribution [14]. The workflow of the wireless channel multipath delay measurement system architecture is shown in Figure 1:

In Figure 1, PC1 (transmitter data processing and control device) oversamples the measurement sequence and processes it through a shaping filter to form a baseband measurement signal. This baseband measurement signal undergoes zero-IF processing and is directly transmitted as an RF signal by the radio transmitter. After traversing the wireless channel, the RF signal forms multipath signals, which are received and stored by the radio receiver. PC2 (receiver control and data processing device) employs Kalman filtering and sliding correlation methods to denoise the received signal and ultimately uses the WRELAX method to solve and analyze the correlated signal, thereby extracting the multipath delay information of the channel.

## 3. Probe Signal Generation

### 3.1. Pseudorandom Noise Sequence Selection

When measuring channels, the first step is to select an appropriate source signal. PN sequences, known for their excellent autocorrelation properties, are commonly used for channel measurements. PN sequences can be divided into binary and non-binary sequences. A typical binary sequence is the m-sequence [15], generated by a Linear Feedback Shift Register (LFSR). Non-binary PN sequences often use the Zadoff–Chu (ZC) [16,17] sequence, which is generated by Equation (1):

(1) $$a_{i}=\left\{\begin{array}{c} e^{j\frac{Z\pi }{N_{m}}{\left(k+i\right)}^{2}},N_{m}\;\mathrm{is}\;\mathrm{Odd}\\ e^{j\frac{Z\pi }{N_{m}}\left[\left(k+i\right)\left(k+i+1\right)\right]},N_{m}\;\mathrm{is}\;\mathrm{Even} \end{array}\right.$$

In Equation (1), $N_{m}$ represents the number of symbols in the sequence, *Z* and $N_{m}$ are coprime, and *k* is any integer.

**Figure 2:**
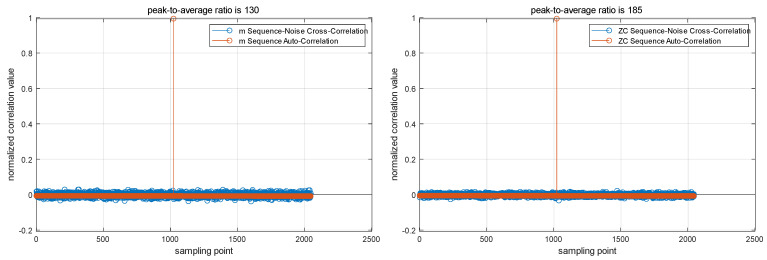
In the measurement simulation system structure diagram, under the same signal-to-noise ratio, performing autocorrelation on both the m-sequence and the ZC-sequence and comparing their peak-to-average ratios allows for a direct observation of their noise resistance performance.

To compare the noise resistance of two types of sequences, their Peak-to-Average Power Ratio (PAPR) is compared under additive white Gaussian noise with a SNR of 10 dB, as shown in Figure 2.

Figure 2 displays the PAPR of both sequences under identical conditions. Although the PAPR of the ZC sequence is higher than that of the m-sequence, the difference is marginal. In addition, the ZC sequence has only one code type within the same sequence length, it cannot be used for simultaneous multi-device measurements, and its generation is complex. In contrast, the m-sequence is simple to generate and can produce multiple sequences of the same length with different initial states, making it suitable for multi-device measurements at the same time. Therefore, this paper selects the m-sequence as the source signal.

### 3.2. Linear Feedback Shift Register Generates M-Sequence

**Figure 3:**
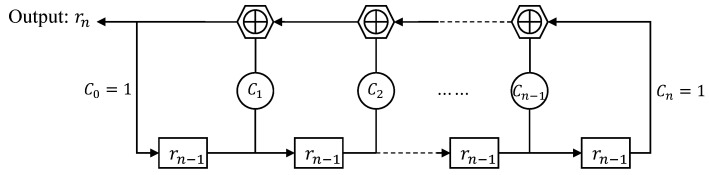
Linear feedback shift register generates m-sequence.

The m-sequence generated by an *n*-stage LFSR has a length of $N_{m}=2^{n}-1$. It is obtained by multiplying the value of each bit in the shift register using feedback coefficients, summing them, and then performing modulo-2 arithmetic. The schematic diagram of the linear feedback shift register is shown in Figure 3:

Based on Figure 3, the relationship between the values in the register and the output sequence values can be expressed by Equations (2) and (3):

(2) $$r_{n}^{i}=C_{1}r_{n-1}+C_{2}r_{n-2}+\cdots +C_{n}r_{0}=\sum_{j=1}^{n}C_{j}r_{n-j}\left(\mathrm{mod}2\right)$$

(3) $$m_{i}=2\cdot r_{n}^{i}-1,i=0,1,2,\cdots ,N_{m}-1$$

Equation (2) is the feedback logic function of the shift register, where $r_{n}^{i}$ represents the output value of the sequence after the i-th shift. To realize the autocorrelation properties of the shift register, the binary sequence output $r_{n}^{i}\left[0,1\right]$ needs to be converted into a bipolar sequence of values $m_{i}\left[\begin{array}{c} -1,1 \end{array}\right]$ using Equation (3), thereby generating the m-sequence $\left\{m_{0},m_{1},\cdots ,m_{N_{m}-1}\right\}$, where $N_{m}$ represents the length of the m-sequence.

### 3.3. Oversampling and Shaping Filtering

The m-sequence that has been generated is oversampled by a factor of *M* to obtain the corresponding oversampled sequence. However, due to the limited bandwidth of radio equipment for transmitting signals, the signal, after oversampling and thereby having an excessively large bandwidth, cannot be transmitted directly via RF. It is necessary to process the oversampled signal through a shaping filter [18].

(4) $$N=N_{m}M$$

(5) $$M_{k}=m_{p},p=\left[k/M\right],k=0,1,\ldots ,N-1$$

In Equation (4), *N* represents the product of the m-sequence length $N_{m}$ and the oversampling rate *M*, which is the length of the signal after oversampling. The symbol $\left[\begin{array}{c} \cdot \end{array}\right]$ denotes the rounding operation. The bipolar m-sequence generated in Equation (3), after processing through Equation (5), yields the oversampled sequence $\{M_{0},M_{1},\cdots ,M_{k},\cdots ,$$M_{N-1}\}$. To form the baseband detection signal, a root-raised cosine filter is used to filter the oversampled signal. The filter specifications include a roll-off factor of 0.25, a filter order of 128, a scaled passband using a Hamming window, a sampling frequency ($F_{s}$) of 110 MHz, and a cutoff frequency ($F_{c}$) of 20 MHz. This sequence, after being processed by a root-raised cosine filter, becomes the baseband signal $\left\{x_{0},x_{1},\cdots ,x_{N-1}\right\}$ for radio transmission.

## 4. Signal Reception Processing

### 4.1. Multipath Signal Reception

After being received through RF, the received signal is demodulated by the radio receiving equipment and converted into discrete data through an ADC to be processed by a PC. Thus, in the received wireless signal sequence $\left\{s_{0},s_{1},\cdots ,s_{N-1}\right\}$, the *k*th symbol $s_{k}$ is shown in Equations (6) and (7):

(6) $$s_{k}=\sum_{i=1}^{L}a_{i}x_{(N+k-n_{i}^{\tau })\mathrm{mod}N}+e_{k}$$

(7) $$n_{i}^{\tau }=\tau_{i}\cdot f_{s}$$

In Equation (6), $S_{(N+k-n_{i}^{\tau })\mathrm{mod}N}$ represents the $\left[(N+k-n_{i}^{\tau })\mathrm{mod}N\right]$th symbol in the transmitted wireless signal sequence, $a_{i}$ represents the channel attenuation factor corresponding to the *i*th path, $n_{i}^{\tau }$ represents the delay of the *i*th path, $f_{s}$ is the upsampling rate, $n_{i}^{\tau }$ represents the number of points of the delay in the discrete domain, and $e_{k}$ is the aliased noise in the received signal, originating from white noise in free space or other interference signals.

### 4.2. Kalman Filtering and Sliding Correlation

For a set of received signals, the down-converted signals are affected not only by multipath superimposed signals but also by the noise $e_{k}$ described in Equation (6). The Kalman filter [19,20], as an efficient recursive filter, estimates the true state measurement value based on the values of each measurement at different times, combined with the results of the previous state and the current state measurement value, and has a good real-time performance and noise reduction effect. In time delay estimation algorithms, the  SNR and real-time performance have a significant impact on the accuracy of time delay estimation. Therefore, one-dimensional Kalman filtering is used to denoise the received aliased signals.

In the time domain, $n^{s}$ groups of wireless signal sequences $\left\{s_{0},s_{1},\cdots ,s_{N-1}\right\}$ are received, and then one-dimensional Kalman filtering is applied to *N* symbols in $n^{s}$ groups, which can be implemented using Algorithm 1.
**Algorithm 1** Implementation of Kalman Filtering**Input:** data: Received Multiple Signal Sets, *m*: Number of Signal Sets, *n*: Signal Length**Output:** signal: Filtered Signals1:**function** kalmanfilter(data,m,n)2:    **for** i=1 to *n* **do**3:        s←data[1:m,i]4:        D←15:        k←16:        x[k]←s[k]7:        A←eye(D)8:        P←eye(D)9:        Q←c∗eye(D)10:      R←d11:      **for** k=2 to *m* **do**12:           x[k]←A∗x[k−1]13:           $P\leftarrow A*P*A^{⊤}+Q$14:           H←eye(D)15:           $K\leftarrow P*H^{⊤}/\left(H*P*H^{⊤}+R\right)$16:           x[k]←x[k]+K∗(s[k]−H∗x[k])17:           I←eye(D)18:           P←(I−K∗H)+P19:        **end for**20:        signal[i]←x[m]21:    **end for**22:**end function**

The filtering process of the Kalman filter is divided into two parts: the time measurement equation and the state update equation. The input parameters for Algorithm 1 of the Kalman filter are the multiple sets of received signals, where the number of signal sets is equivalent to $n^{s}$ and the signal length is equivalent to *N*. In the above algorithm, *D* represents the filtering dimension of the Kalman filter; *k* represents the group time of the symbol; A is the state transition matrix; P represents the estimated covariance; Q is the error between the state transition matrix and the actual process, which needs to be adjusted according to the actual situation; and R is the measurement noise covariance, which can be obtained from observations.

The time measurement equation is implemented in lines 12 and 13, which provide the prior state estimate and the prior estimate covariance at time *k*, respectively. The state update equation is reflected in lines 15, 16, and 18, where the prior estimates are combined with new measurement variables to construct the improved posterior estimate. After the filtering process is completed, the output of the last updated state result is ${\hat{s}}_{k}$, which results in the filtered output signal $\left\{{\hat{s}}_{0},{\hat{s}}_{1},\cdots ,{\hat{s}}_{N-1}\right\}$.

The sliding correlation method is used to measure multipath delays in channels due to its low computational complexity, which provides good real-time capabilities [21,22]. During the channel measurement process, it can display in real time the effects of multiple paths captured within the same time window. The data, after being filtered, are processed using a sliding correlation:

(8) $$r_{xs}\left(k\right)=\sum_{n=0}^{N-1}x_{n}{\hat{s}}_{n+k},k=0,1,\ldots ,N-1$$

(9) $$r_{xx}\left(k\right)=\sum_{n=0}^{N-1}x_{n}x_{n+k},k=0,1,\ldots ,N-1$$

In this context, $x_{n}$ represents the *n*th symbol of the baseband probe signal sequence $\left\{x_{0},x_{1},\cdots ,x_{N-1}\right\}$, ${\hat{s}}_{n+k}$ is the (n+k)th symbol after Kalman filtering, $r_{xs}\left(k\right)$ is the *k*th cross-correlation result within the sliding window *N*, and $r_{xx}\left(k\right)$ is the *k*th autocorrelation result within the sliding window *N*. So, $r_{xs}\left(k\right)$ is obtained by summing the point-by-point products of $\left\{x_{0},x_{1},\ldots ,x_{N}\right\}$ and $\left\{{\hat{s}}_{k},{\hat{s}}_{k+1},\ldots ,{\hat{s}}_{N-1},{\hat{s}}_{0},\ldots ,{\hat{s}}_{k-1}\right\}$. The calculation method of $r_{xx}\left(k\right)$ is the same as that of $r_{xs}\left(k\right)$.

### 4.3. Channel Parameter Extraction Using the WRELAX Algorithm

The WRELAX algorithm is a method of parameter estimation that minimizes a nonlinear least squares cost function [23]. It can decompose multidimensional parameter estimation problems into several one-dimensional estimation issues. The estimation of the overall superimposed signal delays can be accomplished through a series of weighted Fourier transforms and iterative calculations.

(10) $$C_{1}\left({\left\{a_{i},\tau_{i}\right\}}_{i=1}^{L}\right)=\sum_{k=-N/2}^{N/2-1}{\left|R_{xs}\left(k\right)-R_{xx}\left(k\right)\sum_{i=1}^{L}a_{i}\exp\left(-j\frac{2\pi \tau_{i}k}{N}\right)\right|}^{2}$$

In Equation (10), $R_{xx}\left(k\right)$ and $R_{xs}\left(k\right)$ represent the Fourier transform forms of the sliding correlation results $r_{xx}\left(k\right)$ and $r_{xs}\left(k\right)$, respectively. Before applying the WRELAX algorithm, preparations need to be made:

(11) $$R_{xs}={\left[R_{xs}\left(-N/2\right),R_{xs}\left(-N/2+1\right),\cdots ,R_{xs}\left(N/2-1\right)\right]}^{T}$$

(12) $$R_{xx}=diag\left\{R_{xx}\left(-N/2\right),R_{xx}\left(-N/2+1\right),\cdots ,R_{xx}\left(N/2-1\right)\right\}$$

$R_{xs}$ and $R_{xx}$ are the correlation matrix containing multipath information and the original autocorrelation matrix, respectively. Then, the existing delay parameters are extracted from the matrices in the form of delay intervals set in Equation (13).

(13) $$\alpha \left(\tau_{i}\right)=\left\{\exp\left[-j\frac{2\pi \tau_{i}k}{N}\left(-N/2\right)\right],\exp\left[-j\frac{2\pi \tau_{i}k}{N}\left(-N/2+1\right)\right],\cdots ,\exp\left[-j\frac{2\pi \tau_{i}k}{N}\left(N/2-1\right)\right]\right\}$$

(14) $$R_{xs}^{i}=R_{xs}-\sum_{j=1,j\neq i}^{L}{\hat{a}}_{i}R_{xx}\hat{\alpha (\tau_{i})}$$

Based on the aforementioned preparatory formulas, $C_{1}\left({\left\{a_{i},\tau_{i}\right\}}_{i=1}^{L}\right)$ in Equation (10) can be expressed as follows:

(15) $$C_{2}\left(a_{i},\tau_{i}\right)={∥R_{xs}^{i}-a_{i}R_{xx}\alpha \left({\hat{\tau }}_{i}\right)∥}^{2}$$

In Equation (15), ${∥\cdot ∥}^{2}$ is represented as the 2-norm of a matrix. Therefore, the extraction of estimated parameters can be transformed into minimizing ${\hat{\tau }}_{i}$:

(16) $${\hat{\tau }}_{i}=\arg\max_{\omega_{i}}{\left|\alpha^{H}\left(\tau_{i}\right)\left(R_{xx}^{*}R_{xs}^{i}\right)\right|}^{2}$$

Then, derive the corresponding parameters ${\hat{a}}_{i}$:

(17) $$\hat{a_{i}}=\frac{a^{H}\left(\tau_{i}\right)\left(R_{xx}^{*}R_{xs}^{i}\right)}{{∥R_{xx}∥}_{F}^{2}}{|}_{\tau_{i}={\hat{\tau }}_{i}}$$

With the aforementioned preparations, the solution process for the superimposed signals can be summarized as follows:

- Step 1: $R_{xs}^{1}=R_{xs}$ then obtain through Equations (16) and (17);
- Step 2: use ${\hat{\tau }}_{1},{\hat{a}}_{1}$ to substitute into Equation (14) to obtain $R_{xs}^{2}$. Then, substitute $R_{xs}^{2}$ into Equations (16) and (17) to get ${\hat{\tau }}_{2},{\hat{a}}_{2}$. Next, use ${\hat{\tau }}_{2},{\hat{a}}_{2}$ to substitute back into Equation (14) to recalculate $R_{xs}^{1}$. Repeat the operations of Step 1 until convergence is achieved, which means the estimated parameters no longer changes;
- Step 3: use the results from Step 2, substitute ${\left\{{\hat{\tau }}_{i},{\hat{a}}_{i}\right\}}_{i=1}^{2}$ into Equation (14) to obtain $R_{xs}^{2}$, then estimate ${\hat{\tau }}_{3},{\hat{a}}_{3}$ by substituting into Equations (16) and (17). Similarly to Step 2, iterate the updating of ${\left\{{\hat{\tau }}_{i},{\hat{a}}_{i}\right\}}_{i=1}^{3}$ until the algorithm converges and the estimated parameters no longer change;
- …… Similarly, when the number of multipath components is *L*;
- Step L: use the results from Step L−1, substitute ${\left\{{\hat{\tau }}_{i},{\hat{a}}_{i}\right\}}_{i=1}^{L-1}$ into Equation (14) to obtain $R_{xs}^{L}$, then estimate ${\hat{\tau }}_{L},{\hat{a}}_{L}$ by substituting into Equations (16) and (17). Continue updating ${\left\{{\hat{\tau }}_{i},{\hat{a}}_{i}\right\}}_{i=1}^{L}$ in a loop similar to Step L−1 until the estimated parameters no longer change.

Additionally, in actual channel measurements, since the number of multipath components in the measurement channel cannot be predetermined, WRELAX can also acquire the number of delay characteristics needed for matched positioning based on the amplitude sizes.

## 5. Simulation Analysis

To verify the performance of the algorithm proposed in this paper, the following simulation experiment was designed: the linear feedback shift register of the m-sequence has an order of n=10; the number of symbols is $N_{m}=1023$; the PN chip is $T_{m}=100$ ns; the sampling rate is $f_{s}=110$ MHz; the sampling interval is $T_{s}=9.1$ ns; the noise type is additive Gaussian noise; the baseband signal bandwidth is set to 20 MHz; and the RF frequency is 1.5 GHz. These parameters were employed to analyze the method’s performance under various conditions.

### 5.1. The Denoising Performance of the Kalman Filter

A set of channel noises ranging from −30 dB to 30 dB was established, and then the SNR of the received signal and the filtered signal was calculated. The results are shown in Table 1.

Table 1 displays the relationship between the SNR of received signals before and after Kalman filtering. It can be observed that although the noise reduction effectiveness decreases as the set SNR of the signal increases, overall, the Kalman filtering method still manages to maintain an improvement of over 6 dB.

### 5.2. Simulation Results

**Figure 4:**
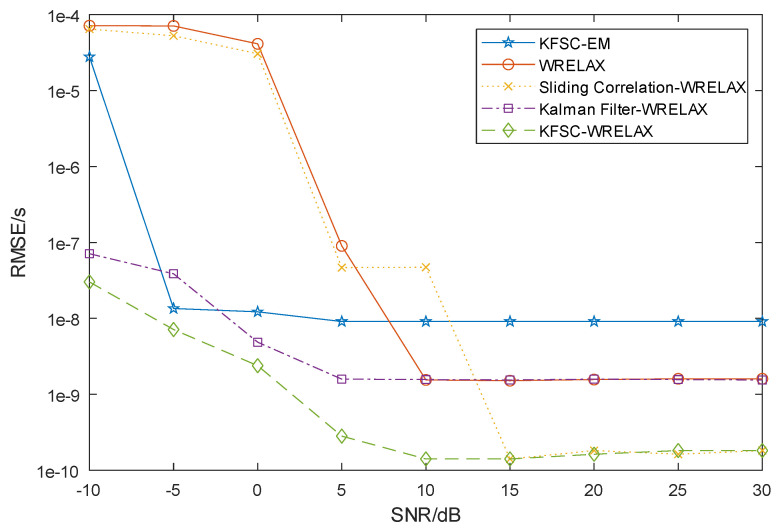
In simulation error analysis diagram, five methods were employed, and multiple simulation validations were conducted while keeping other simulation parameters consistent. By calculating the estimated delay errors for each method and obtaining the average estimated error value, it can be directly observed that under different signal-to-noise ratio conditions, the method proposed in this paper effectively improves the estimation accuracy.

To verify the estimation performance across different methods and under various SNR conditions, conduct independent experiments multiple times under each method and signal-to-noise ratio condition and calculate the root mean square error (RMSE) of the final result. The parameters of the signal source remain unchanged with a multipath number *L* of 2 and corresponding delays are $\tau_{1}=9.1$ ns and $\tau_{2}=54.6$ ns; amplitude attenuation coefficients are $\alpha_{1}=1$ and $\alpha_{2}=0.05$, representing a relative attenuation of 13 dB. In Figure 4, the five curves represent the root mean square error of delay estimation obtained using the following five methods: ‘WRELAX’ (multipath delay extracted directly by the WRELAX algorithm without noise processing of the received signal), ‘Sliding Correlation-WRELAX’ (multipath delay extracted by the WRELAX algorithm after sliding correlation processing of the received signal), ‘Kalman Filter-WRELAX’ (multipath delay extracted by the WRELAX algorithm after Kalman filtering of the received signal), ‘KFSC-WRELAX’ (multipath delay extracted by the WRELAX algorithm after processing by the designed scheme in this paper), and ‘KFSC-EM’ (multipath delay extracted by the EM algorithm after processing by the designed scheme in this paper).

From Figure 4, it is clearly observable that the estimation errors of various algorithms are influenced by noise interference, and overall, the errors decrease as the SNR increases. Observing the curves for ‘WRELAX’, ‘Sliding Correlation-WRELAX’, ‘Kalman Filter-WRELAX’, and ‘KFSC-WRELAX’, it is evident that at low SNR levels the Kalman Filter shows more significant effects in estimation, with estimation errors about 30 dB lower than methods not using the Kalman Filter. At high SNR levels, the sliding correlation shows more pronounced effects in estimation, with errors about 10 dB lower than methods not employing sliding correlation. Comparing the ‘KFSC-EM’ [24,25] and ‘KFSC-WRELAX’ curves, the WRELAX method consistently outperforms the EM algorithm throughout. Thus, it can be seen that the KFSC-WRELAX method performs better than other methods in terms of estimation accuracy.

### 5.3. Channel Simulator Test Results

To verify the effectiveness of the algorithm, this study conducts simulation tests using a channel simulator [26,27], where the RF signal is received after passing through the ZTI-32 channel simulator. The parameters of the channel simulator are configured, and then the algorithm described in this paper is used to extract channel parameters.

The test results from the ZTI-23 channel simulator show that, as indicated in Table 2, even when the time difference between paths is several tens of nanoseconds, the estimated delay error can be controlled within 1 ns, and the number of iterations is also well managed. Additionally, from a theoretical perspective, the greater the amplitude attenuation difference between two paths, the more difficult it is to separate their delays, which in turn results in a relatively higher number of iterations. The results from the channel simulator are consistent with the theory analysis.

### 5.4. Corridor Test Results

**Figure 5:**
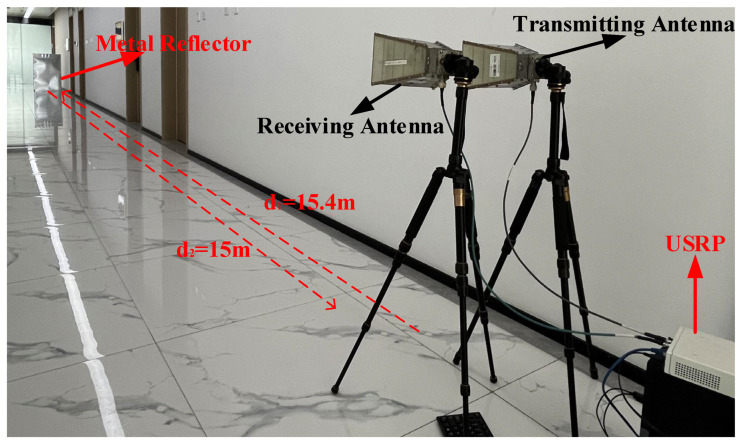
Corridor test environment diagram.

To further validate the feasibility of the proposed approach, this study employed corridor testing [28,29], utilizing directional antennas to control the direction of signal transmission. Reflective paths were created using metal plates and the arrangement placed the transmitting antenna behind the receiving antenna, establishing the first path. By adjusting the distance between the metal reflector and the antennas, the delay of the second path was effectively controlled. The testing environment is illustrated in Figure 5:

In Figure 5, the subtraction and addition of $d_{1}$ and $d_{2}$ represent the transmission distances for the first and second paths, respectively. From this, the delay difference between them is calculated to be 100 ns. Analyzing the signals received by the antenna enables the identification of the sample points where the two paths differ. By calculating the time interval between these sample points, the absolute delay for both paths can be accurately determined. The measurement results are presented in Table 3:

In Table 3, given the reception sampling frequency of 110 MHz and a sampling interval of 9.1 ns, the absolute delay difference between the two paths is calculated to be 100.22 ns, with a total error of 0.22 ns, which corresponds to a measurement distance error of 0.07 m. Based on the sampling interval, the estimation error can be computed as $\left(-T_{s}c/2,T_{s}c/2\right)$ (*c* represents the speed of light, $c=3\times 10^{8}$), or approximately (−1.37 m, 1.37 m). According to the results of the corridor testing, the measurement error falls within the specified range and meets the expected outcomes.

## 6. Conclusions and Discussion

This paper proposes an improved method for measuring multipath delays in wireless channels tailored to environmental needs. This method integrates the correlation properties of m-sequences, Kalman filtering, sliding correlation, and the WRELAX estimation algorithm, providing a high-resolution channel delay estimation algorithm suitable for various noise environments. Kalman filtering enhances the signal-to-noise ratio by more than 6dB, while the sliding correlation method utilizes the autocorrelation properties of PN sequences to identify multipath correlation peaks. The results are then iteratively estimated for wireless channel multipath delay parameters using the WRELAX algorithm. The delay resolution and estimation error of the proposed algorithm outperform other methods to varying degrees under different signal-to-noise ratios. Experimental simulations and channel simulator test results demonstrate that the KFSC-WRELAX method achieves low error-estimation delays while controlling the number of iterations.

## Figures and Tables

**Table 1:** SNR before and after kalman filtering.

Set SNR	−20 dB	−10 dB	0 dB	10 dB	20 dB	30 dB
Post-Filtering SNR	−12.33 dB	−2.76 dB	6.99 dB	16.83 dB	26.71 dB	36.62 dB
SNR Improvement	7.67 dB	7.24 dB	6.99 dB	6.83 dB	6.71 dB	6.62 dB

**Table 2:** Channel simulator test results.

Two-Path Delay Difference	Two-Path Amplitude Attenuation	Measured Delay Difference	Measured Amplitude Attenuation	Number of Iterations
73 ns	−15 dB	72.73 ns	−14.04 dB	26
110 ns	−7 dB	109.82 ns	−7.45 dB	19
110 ns	0 dB	109.09 ns	−0.54 dB	13

**Table 3:** Corridor test results.

First Path: d	Second Path: d	Absolute Delay	Measured Delay Points	Measured Absolute Delay
0.4 m	30.4 m	100 ns	11	100.22 ns

## Data Availability

Data available upon request from the authors if the paper is accepted.

## Abbreviations

The following abbreviations are used in this manuscript:

WRELAX	Weighted Fourier Transform combined with the RELAXation
PN	Pseudorandom Noise
KF	Kalman Filtering
FZC	Frank Zadoff Chu
SNR	Signal-to-Noise Ratio
LFSR	Linear Feedback Shift Register
ZC	Zadoff-Chu
PAPR	Peak-to-Average Power Ratio
RMSE	Root Mean Square Error
